# Supplementary material for: Meteorological and Nutrient Conditions Influence Microcystin Congeners in Freshwaters
Source: Toxins (Basel). 2019 Oct 26;11(11):620. doi: 10.3390/toxins11110620 (PMC6891300; doi:10.3390/toxins11110620)
Supplement: Supplementary file 1 [file toxins-11-00620-s001.zip › toxins-619775 supplementary proofdone.docx]

Supplementary Materials: Meteorological and Nutrient Conditions Influence Microcystin Congeners in Freshwaters

Zofia E. Taranu, Frances R. Pick, Irena F. Creed, Arthur Zastepa and Sue B. Watson


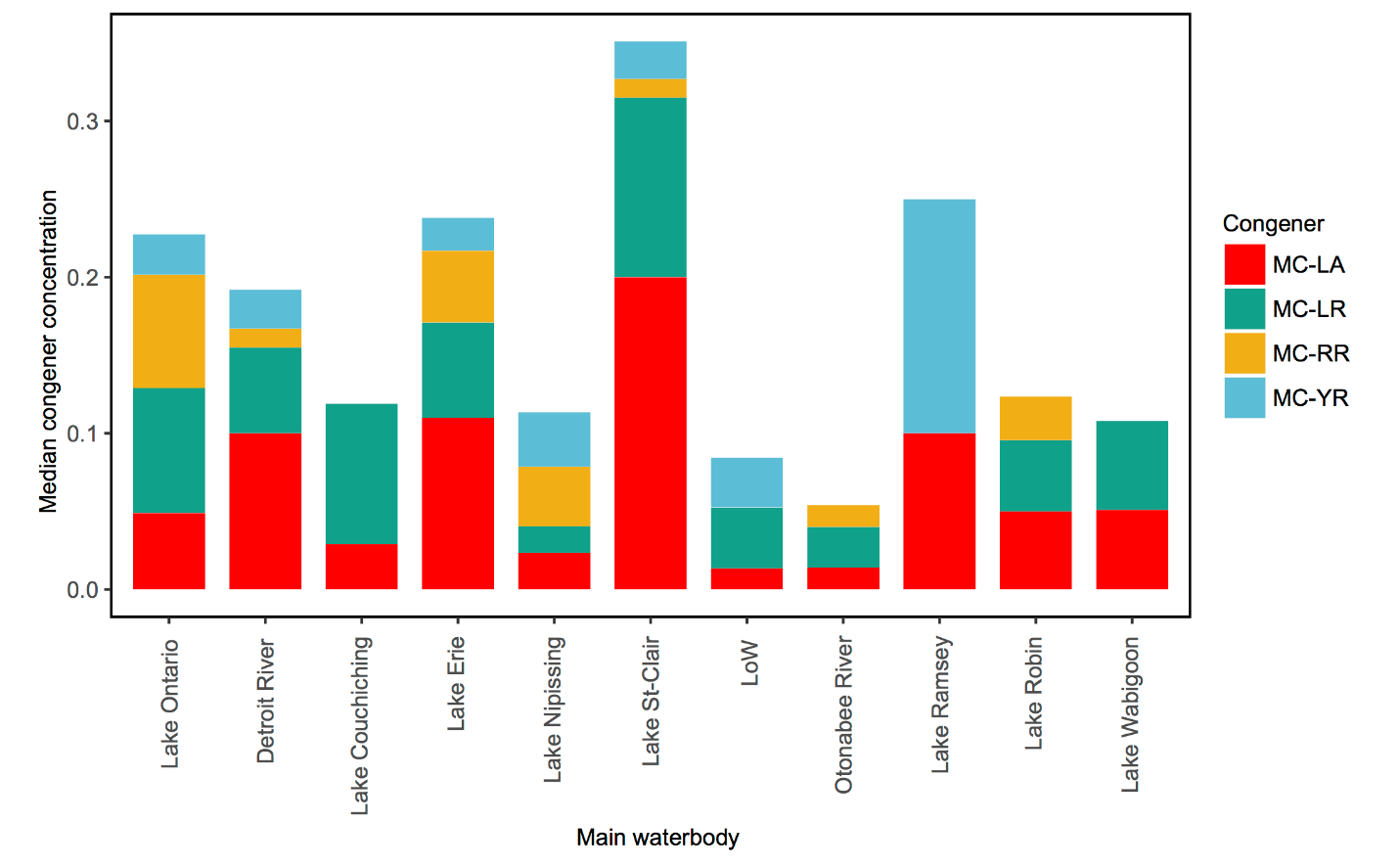


**Figure S1.** Bar plots of average MC-congener concentrations across each major water source (OMECP Great Lakes basin dataset, N America). Temporal change not shown. Averages are based on the median value as opposed to the arithmetic mean given the skewed distribution of the data caused by numerous values near the detection limit.


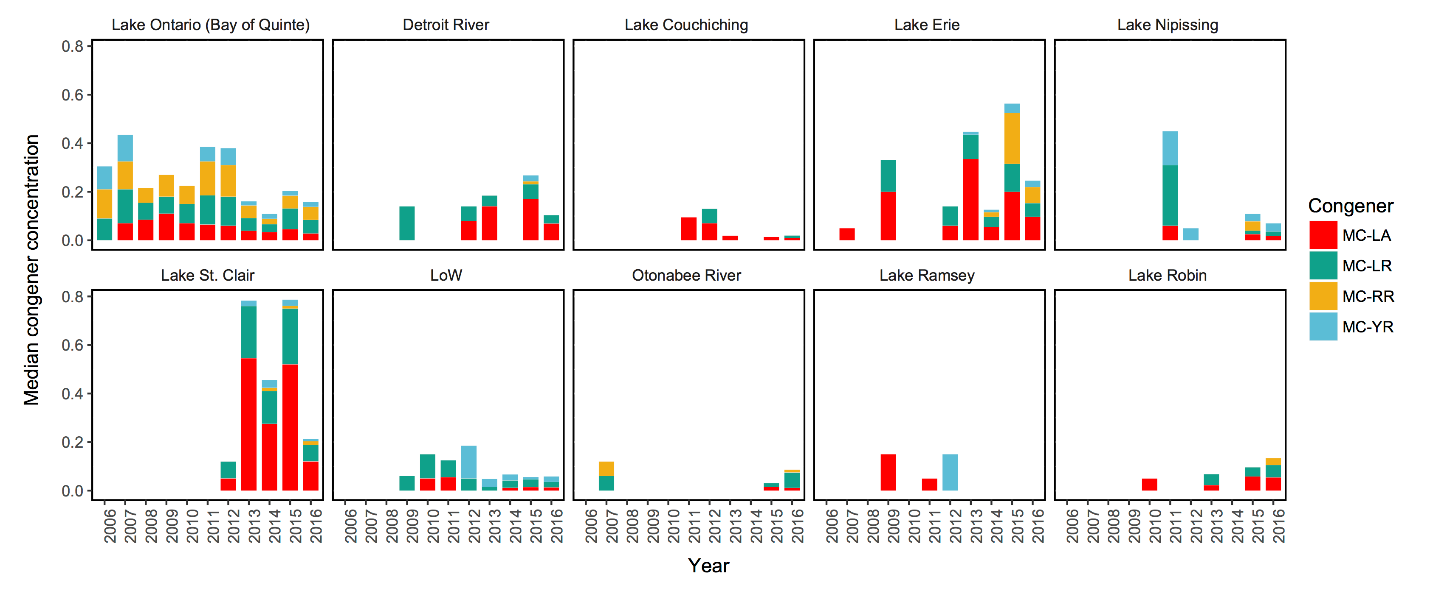


**Figure S2.** Bar plots of yearly averaged (2006–2016) MC congener composition across each Great Lakes basin raw water intake source, illustrating the space-time variability in congener concentrations. Averages are based on the median value as opposed to the arithmetic mean given the skewed distribution of the data caused by numerous values near the detection limit. NB: Lake Wabigoon was only sampled in 2013 and was thus omitted from this figure (please see Figure S1 for the Lake Wabigoon bar plot).


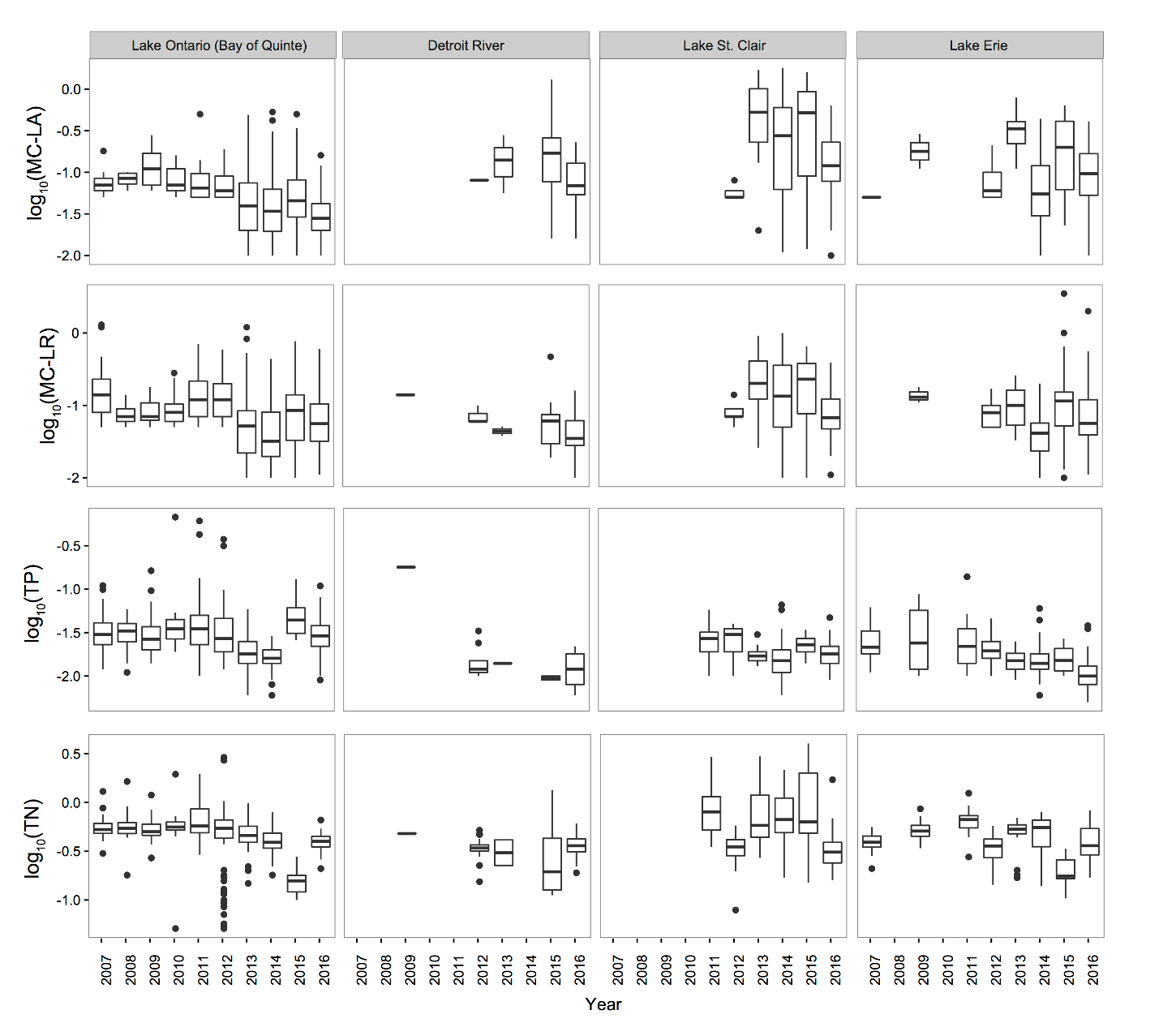


**Figure S3.** Boxplots of concentrations of MC-LA, MC-LR, total phosphorus (TP) and total nitrogen (TN) from the Great Lakes basin water sources most frequently sampled by the OMECP, namely, Lake Erie (Elgin, Essex, Pelee and Union raw water intake sites), Bay of Quinte (Bayside, Belleville, Desronto, and Picton raw water intake sites), Detroit River (City of Windsor intake site) and Lake St. Clair (Lakeshore at Stoney and Lakeshore intake sites). Boxes represent the 1st and 3rd quartiles and solid lines represent the median values.


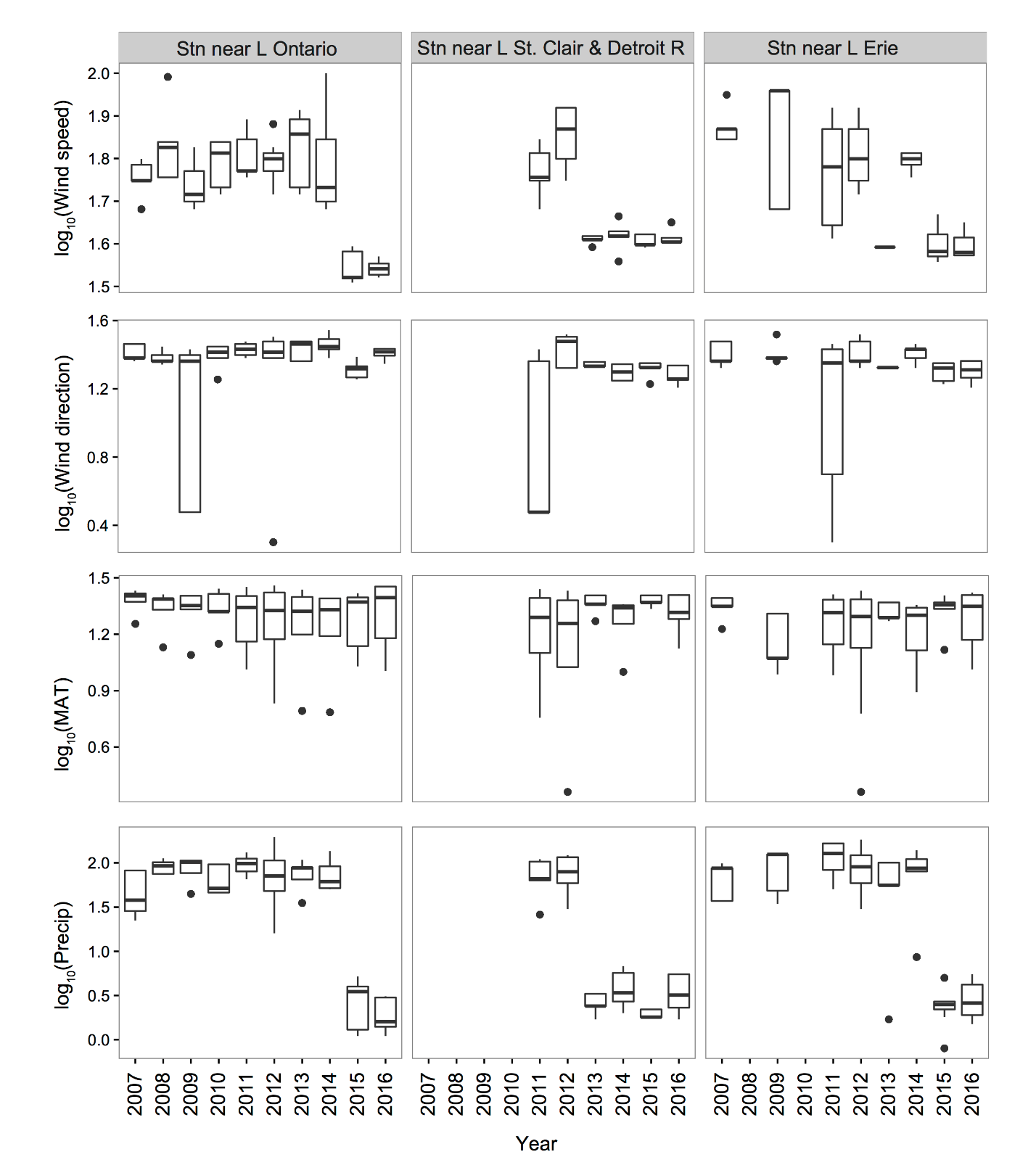


**Figure S4.** Boxplots of weather conditions from nearby weather stations from the most frequently sampled water sources by the OMECP (see Figure S5 for details).


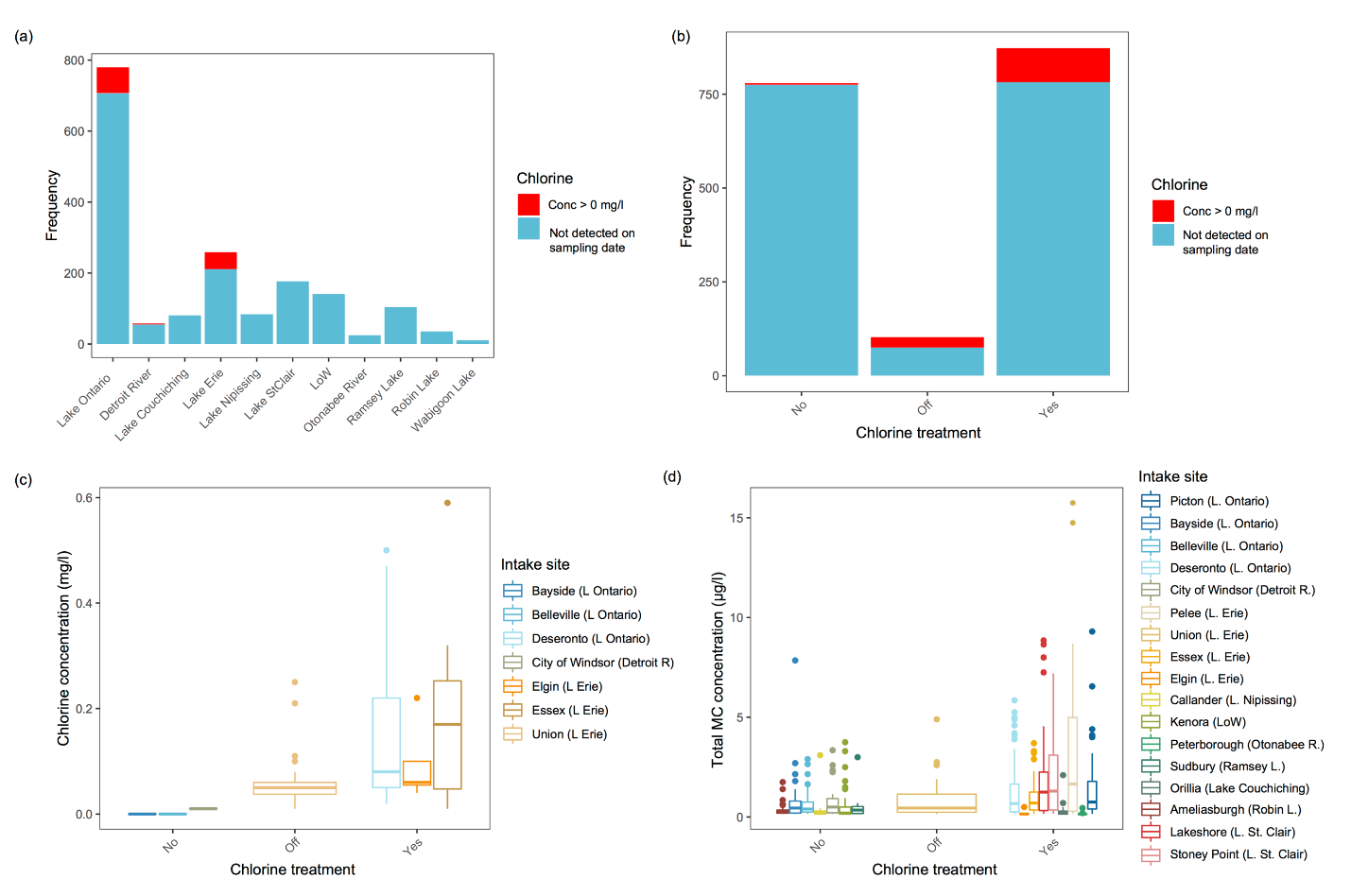


**Figure S5.** Summary of chlorination treatment and chlorine concentration of the OMECP raw water intake sites.


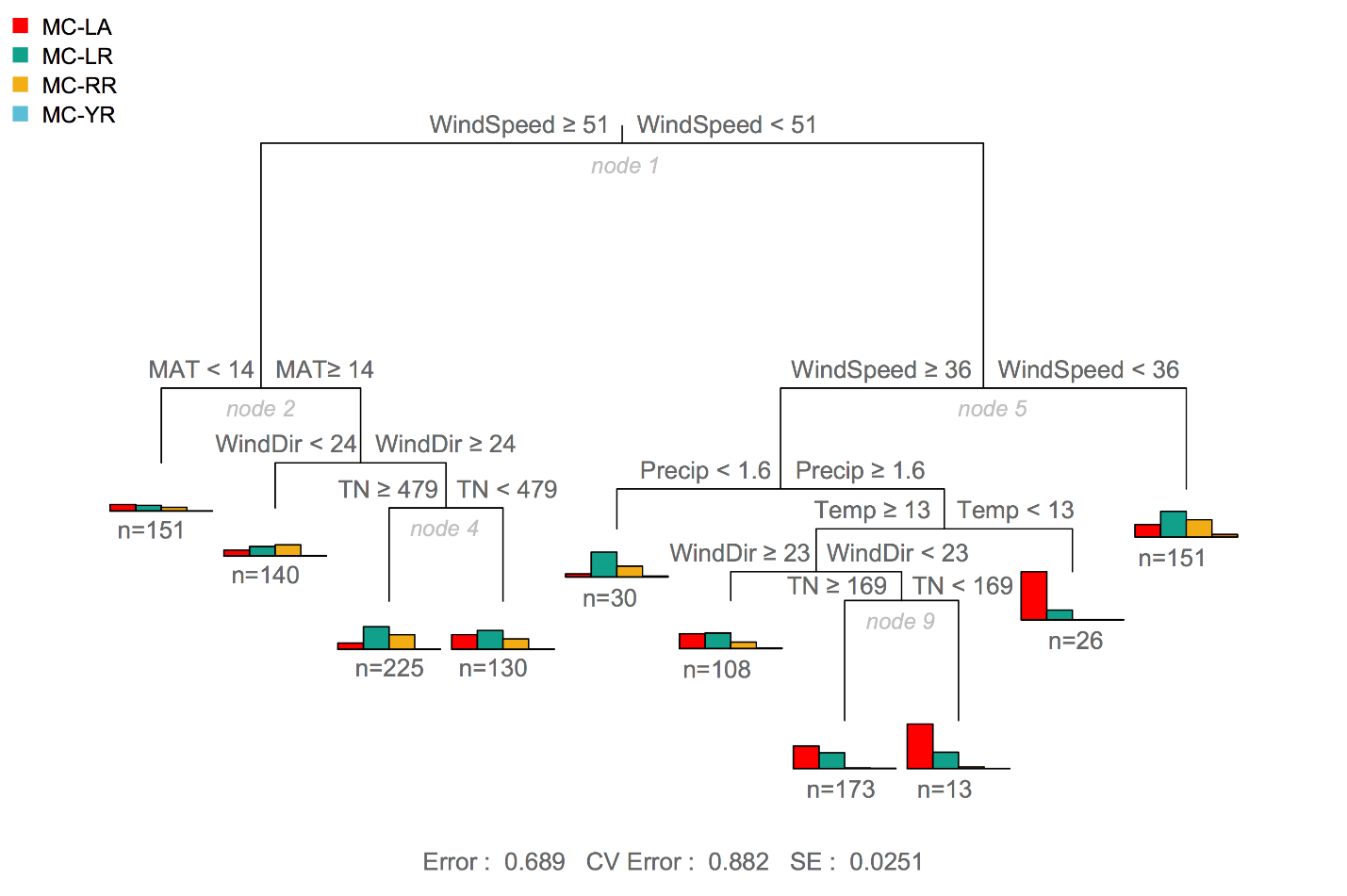


**Figure S6.** Multivariate regression tree (MRT) of congener relative abundance from Great Lakes basin raw water intake data constrained by meteorological conditions (MAT = maximum air temperature, WindSpeed= maximum wind speed, WindDir = direction of wind with the highest. speed, Precip = precipitation) and nutrient concentrations (TN = Total Nitrogen). Note: Total Phosphorus was omitted from this analysis.


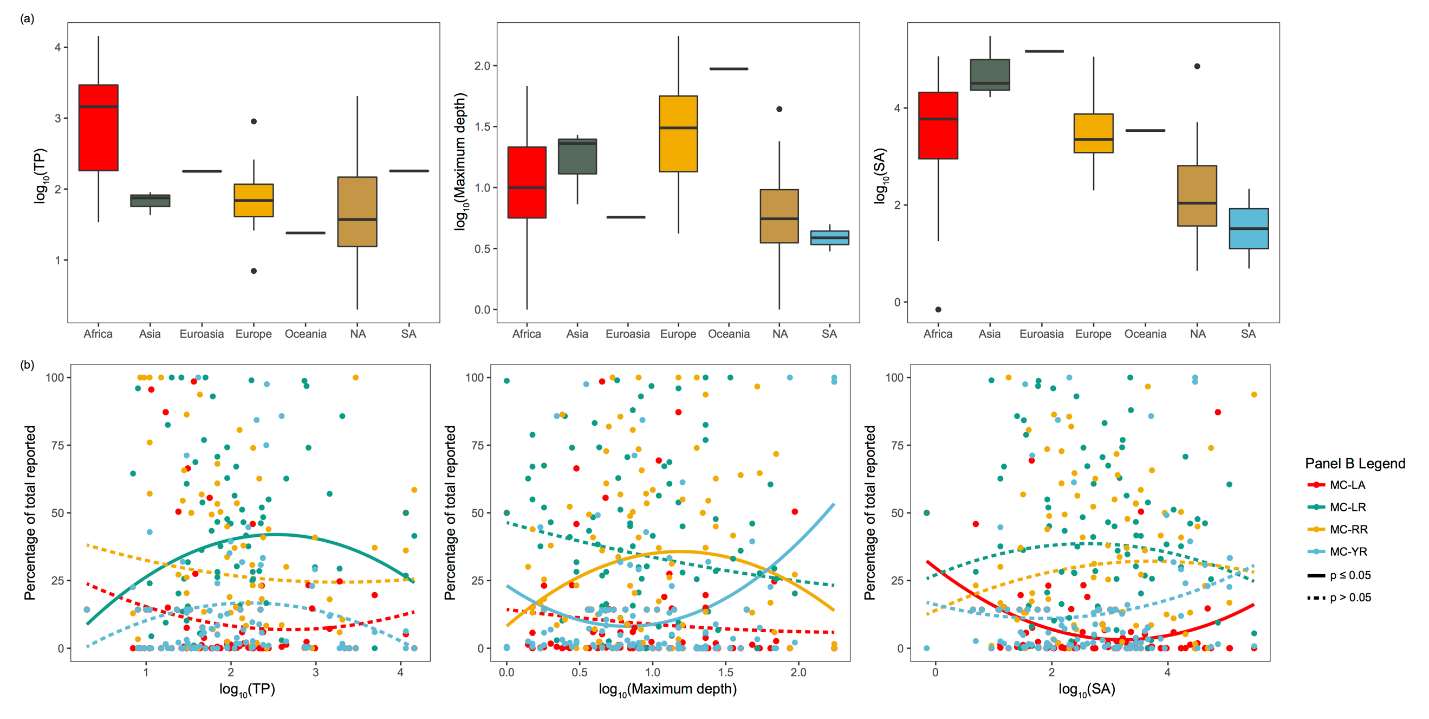


**Figure S7.** Summary of global dataset. (**a**) Boxplot of lake characteristics (average total phosphorus concentration (TP), maximum depth (m) and surface area (ha) of each lake) across continents. (**b**) Relationship between relative abundance of each congener and lake characteristics from global dataset (OMECP raw intake sites were omitted).


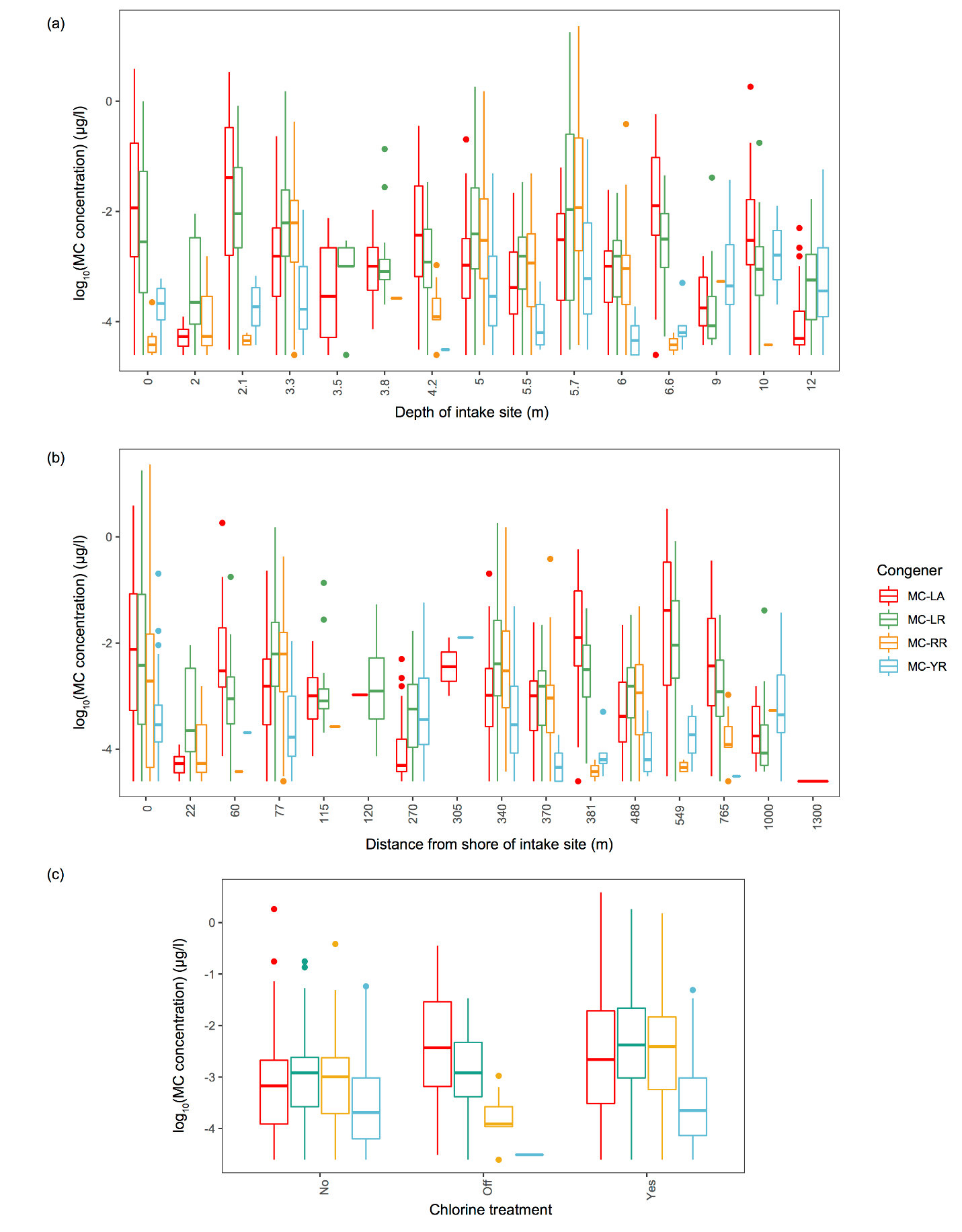


**Figure S8.** Relationship between congener composition and OMECP raw water intake location and water treatment plant chlorination status.


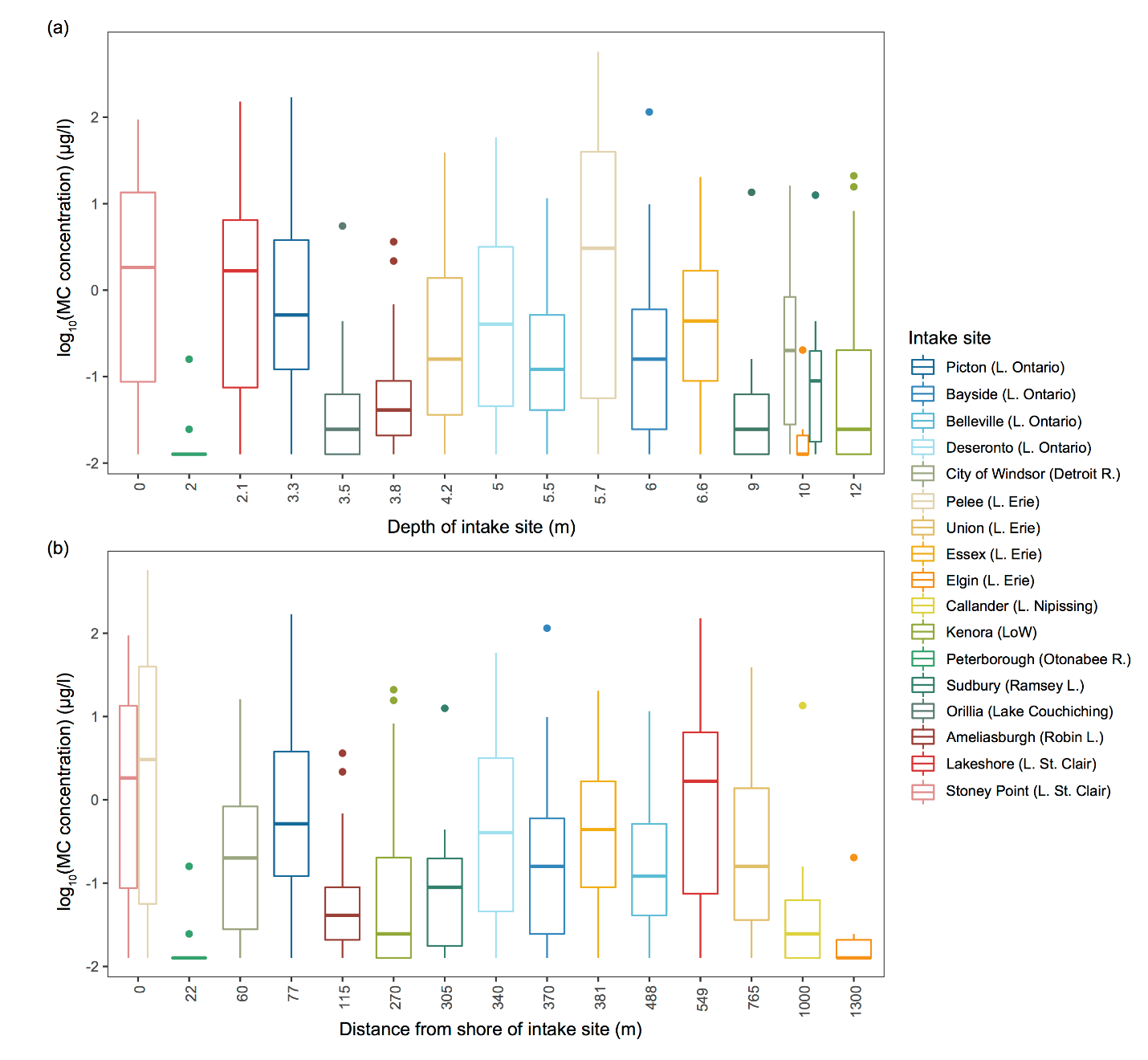


**Figure S9.** Relationship between total MC concentration and location of OMECP raw water intake sites.
